# Colorectal carcinogenesis: an archetype of gut microbiota–host interaction

**DOI:** 10.3332/ecancer.2018.865

**Published:** 2018-09-05

**Authors:** James L Alexander, Alasdair J Scott, Anna L Pouncey, Julian Marchesi, James Kinross, Julian Teare

**Affiliations:** Centre for Digestive and Gut Health, Department of Surgery and Cancer, Imperial College London, 10th Floor QEQM Building, St Mary’s Hospital, South Wharf Road, London W2 1NY, UK

**Keywords:** colorectal cancer, microbiota, fusobacterium, metabolic function, interaction

## Abstract

Sporadic colorectal cancer (CRC) remains a major cause of worldwide mortality. Epidemiological evidence of markedly increased risk in populations that migrate to Western countries, or adopt their lifestyle, suggests that CRC is a disease whose aetiology is defined primarily by interactions between the host and his environment. The gut microbiome sits directly at this interface and is now increasingly recognised as a modulator of colorectal carcinogenesis. Bacteria such as *Fusobacterium nucleatum* and *Escherichia coli* (*E. Coli*) are found in abundance in patients with CRC and have been shown in experimental studies to promote neoplasia. A whole armamentarium of bacteria-derived oncogenic mechanisms has been defined, including the subversion of apoptosis and the production of genotoxins and pro-inflammatory factors. But the microbiota may also be protective: for example, they are implicated in the metabolism of dietary fibre to produce butyrate, a short chain fatty acid, which is anti-inflammatory and anti-carcinogenic. Indeed, although our understanding of this immensely complex, highly individualised and multi-faceted relationship is expanding rapidly, many questions remain: Can we define friends and foes, and drivers and passengers? What are the critical functions of the microbiota in the context of colorectal neoplasia?

## Introduction

By recent estimates, almost a quarter of a million Europeans are diagnosed with colorectal cancer (CRC) annually, and incidence is rising [1]. Perhaps, more concerning, since 1998, in contrast to the declines seen in older patients, younger adults have experienced an apparent increase in the incidence of CRC [2]. Indeed, although CRC is predominantly a disease of later age in the West (median age at diagnosis approximately 70 years [3]), the age-specific relative risk has been increasing in younger generations over recent decades, meaning that adults born in the USA in 1990 have double the risk of colon cancer and quadruple the risk of rectal cancer compared to those born in 1950 [4].

Traditional models of cancer aetiology have focussed on understanding how mammalian genetic susceptibility combines with risk factors such as smoking to drive carcinogenesis. As a contributor to disease pathogenesis, the gut microbiota (namely, the bacteria, viruses, archaea and eukaryotic organisms that inhabit the human gastrointestinal tract) had largely hidden in the blind spot of the medical research community until the last 10 years. This was fundamentally a technological issue: the culture-based methods previously used to study bacteria (still the mainstay of clinical microbiology laboratories) are not well suited to the large-scale analysis of the cornucopia microorganisms present in the large intestine. But with the advent of next-generation sequencing, there has been a paradigm shift in our ability to catalogue and characterise this ecosystem [5]. There has followed a rapid proliferation of interest in this so-called ‘forgotten organ’ and the development of the Human Microbiome Project, which attempted to database the microbiota in much the same way that geneticists had done with the human genome years earlier [6]. In this review, we discuss how the rapid development in the knowledge of the gut microbiome is providing novel insights into colorectal carcinogenesis.

## Diet, lifestyle, the gut microbiome and CRC

CRC rates vary by up to 10-fold around the world. The majority of cases and deaths occur in countries with high or very high human development indices [7]. Low- and middle-income countries transitioning quickly towards a more westernised society and economy exhibit the starkest increases in incidence [8]. Examples include Brazil and Bulgaria, with average annual percentage changes in the incidence of 7.2 and 3.6, respectively [7]. Given the rapidity of this trend, there is a compelling argument for the pre-eminent contribution of lifestyle factors over host genetics in the pathogenesis of this disease. Indeed, family history of CRC accounts for only a small proportion of the variation [9]. Established risk factors include alcohol [10], lack of physical activity [11], smoking [12], obesity [13] and perhaps most importantly, diet.

The observation that diet might influence the risk of gastrointestinal diseases such as CRC is historic—Burkitt made the link to dietary fibre depletion in the 1970s [14]; although the existing data with regard to fibre intake are somewhat conflicting [15, 16]. There is now a body of evidence pointing to high intake of red and processed meat as an integral player in CRC risk [17–20].

Compelling evidence for diet’s importance comes from migrant studies which have shown that within one generation, immigrant populations adopt the colon cancer incidence of the host Western population [21]. Despite this observation, genome-wide association studies have required very large cohorts of subjects to demonstrate the significant linkage between loci that influence CRC risk and diet, suggesting that another factor is at play [22, 23]. The microbiota have been implicated in the metabolism of red meat derivatives and the consequent production of choline, trimethylamine and hydrogen sulphide, all of which may be deleterious to host health [24–26]. Much work has centred on the microbial metabolism of fibre, which is relatively deficient in the Western diet [27]. There is considerable evidence that fibre fermentation products, such as the short chain fatty acids (SCFAs), which are essential energy sources for normal colonocytes, also dampen inflammatory processes and are anti-carcinogenic [28, 29]. For example, one such SCFA, butyrate, has been shown to down-regulate the canonical Wnt-signalling pathway [30], inhibit cancerous colonocyte proliferation through histone deacetylase inhibition [31] and induce apoptosis [32]. Studies have shown a reduction in butyrate-producing phyla in the microbiomes of animal models and patients with CRC [33–35].

Studies comparing African Americans and rural Africans perhaps best support the role of the gut microbiota as a mediator of diet-induced cancer risk [36]. The work of O’Keefe and co-workers demonstrated that the diets of African Americans living in Pittsburgh were dominated by fat and processed meat, in contrast to those of rural South Africans, which were dominated by fibre [37]. African Americans, who are at high risk of CRC, have colons predominated by Prevotella species, while rural Africans, with low risk of CRC, are colonised by Bacteroides species. When these diets were switched, rapid reciprocal shifts in the microbiota and metabolomes of the groups were accompanied by marked changes in mucosal biomarkers of cancer risk. The conclusion from this work is that a change in bacterial co-occurrences across niche-specific microbial networks associated with symbiotic metabolism of dietary nutrients pre-conditions the gut into either a pro-oncogenic or protective state. The relative absence of *Fusobacterium nucleatum* (an organism with links to CRC which we will discuss in greater detail) in CRCs from patients with high-fibre diets supports this hypothesis [38].

In addition to dietary patterns, migration changes many other aspects of the environment and there is now evidence that the microbiome is at least modified by CRC risk factors such as smoking [39, 40], exercise [41, 42] and alcohol [43]. There is also evidence that differences in host genotype affect the carbohydrate landscape of the distal gut and these in turn interact with the diet to alter the composition and function of resident microbes in a diet-dependent manner. Therefore, it is possible that patients genetically predisposed to CRC have a modified metabolically active microbiome, which is not only determined by their genes but also by their family environment, dietary habits and lifestyle choices.

## Inflammation, obesity, CRC and the microbiota

A unifying issue in the pathogenesis of CRC is the presence of persistent low-grade inflammation. This is supported by evidence that cancer risk may be reduced by greater than a quarter by anti-inflammatory drugs [44]. A large proportion of morbidly obese individuals (30%–70%) gets CRC, and obesity is characterised by systemic low-level inflammation [45]. Metataxonomic studies have demonstrated elevated numbers of *Firmicutes* and decreased levels of *Bacteroidetes* and a reduced microbial diversity and genetic abundance in obese individuals [46]. It is therefore feasible that cancer in the colon is driven by particular microbes that are fostered in an obese-related inflammatory environment.

Bariatric surgery presents a useful opportunity for studying the impact of the gut microbiota on host metabolic function, and gastric bypass surgery has a profound impact on the distal microbiome construct and its metabolic function [47, 48]. It is interesting to note, therefore, the paradox in patients undergoing bariatric surgical procedures, where epidemiological evidence suggests the risk of CRC in fact increases [49] and patients have poorer outcomes [50]. Evidence from post gastric bypass murine models has also demonstrated that the faecal stream, which is partly a product of microbiota-mediated metabolism of dietary substrates, is highly genotoxic [51]. This suggests that the relationship between obesity and CRC may be modulated by the microbiota, and that surgical bariatric interventions may inadvertently alter an individual’s CRC risk by driving the microbiota towards a pro-carcinogenic state.

## The case for an oncogenic driver species in CRC

The concept that microbes might be pathogenic mediators of neoplasia is, of course, not novel. In the 1980s, *Helicobacter pylori* was identified in the stomach of patients with gastritis and peptic ulceration, providing a link between the bacterium and gastric cancer [52]. The initial hostile response of the scientific community to this, now well-established, association is perhaps instructive [53]. The human papillomavirus virus is relatable to the vast majority of cervical cancers, as well as some anal, vulvar and oropharyngeal neoplasms [54]. Liver fluke infections are strongly associated with cholangiocarcinoma in East Asia [55, 56] and chronic infection with the parasite *Shistosoma haematobium* predisposes carriers to urothelial malignancy [57]. With the colon hosting the great majority of microbiota residing in the human body [58], it is only logical that investigators have sought to make similar discoveries with regard to CRC.

**Figure 1.**
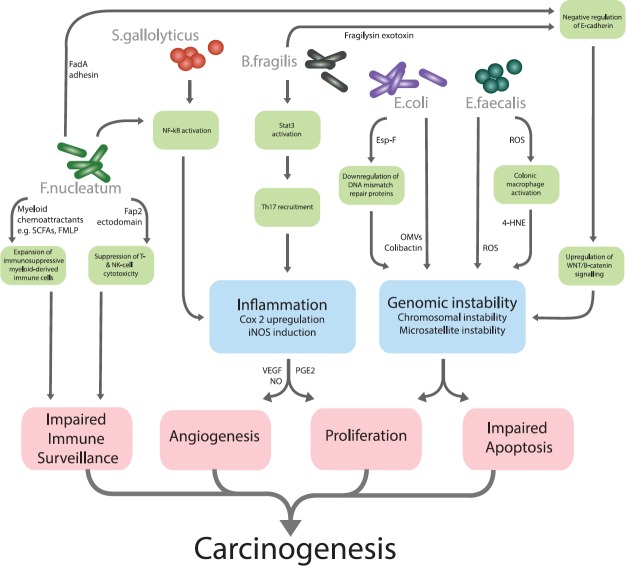
Gut microbiota and their mechanistic links to colorectal carcinogenesis.

With the examples from other body sites in mind, research to date on the gut microbiota has attempted to define specific microbial candidates that serve as ‘alpha bacteria’ or pro-oncogenic driver species in CRC [59]. On this basis, the evidence is now available for a stable of bacteria with mechanistic plausibility as aetiological agents in CRC (Table 1 and Figure 1).

The gram-negative anaerobe *Fusobacterium nucleatum* has been associated with carcinomas and adenomas of the colon and rectum in a number of human studies [60–65]. *F. nucleatum* was typically regarded as an oral commensal bacterium and has a relatively low abundance in the healthy human colon [66]. It has long been recognised as one of the principal pathogens in gingivitis and periodontitis, and it is from this setting that biologically plausible mechanisms of colorectal oncogenesis first emerged [67]. In an *in vitro* model, its fadA adhesin was shown to bind to E-cadherin on CRC cell lines resulting in invasion by the organism and activation of the β-catenin/Wnt signalling cascade with consequent stimulation of cell proliferation [68]. Furthermore, fadA gene and protein expression were increased in adenomas and adenocarcinomas compared to healthy mucosa from non-tumorous individuals, and correlated with significantly raised expression of Wnt genes, consistent with the *in vitro* data.

Further support for a tumorigenic role of *F. nucleatum* is provided by a series of experiments in which Apc^Min/+^ mice, which develop gastrointestinal tumours, were exposed to a *F. nucleatum* strain isolated from a patient with inflammatory bowel disease. Exposure resulted in a significant increase in the numbers of colonic tumours and, in accord with human data, *F. nucleatum* was enriched in tumour tissue relative to adjacent normal mucosa [61]. Interestingly, *F. nucleatum* did not induce colitis in the Apc^Min/+^ mice, which contrasts with the known mechanism of accelerated tumorigenesis induced by enterotoxigenic *B. fragilis* in the same model. Rather, the authors found that *F. nucleatum* recruited tumour-permissive immune cells to the murine tumour microenvironment, which potentiated tumour progression. Similarly, the expression of relevant immune cell marker genes positively correlated with *Fusobacterium* spp*.* abundance in human CRC specimens. In another study, the *F. nucleatum* Fap2 ectodomain inhibited human natural killer (NK) cell anti-tumour toxicity via the TIGIT receptor which is expressed on all T cells, permitting evasion of tumour cells from immune surveillance [69]. *F. nucleatum* has also been shown to activate TLR-4 signalling to MYD88, leading to activation of Nuclear Factor-kB via increased expression of microRNA21 (miR21) [70]. Collectively, these experimental data support an association between *F. nucleatum* and human CRC and provide plausible mechanisms for promoting oncogenesis directly, via activation of Wnt and, indirectly, by dampening of host anti-tumour immune responses.

Importantly, there is now evidence from human populations that the presence of *Fusobacteria* may be of prognostic importance, as it is associated with CIMP positivity, TP53 wild-type, hMLH1 methylation positivity, MSI and CHD7/8 mutation positivity [71]. Mima and co-workers found that *F. nucleatum* was associated with a lower density of CD3+ T cells, which have an important role in the anti-CRC adaptive immune response, conferring better prognosis [72]. The same investigators also found that the amount of *F. nucleatum* DNA in CRC tissue is associated with shorter patient survival [73] and intriguingly, there is evidence emerging that the organism persists and migrates in metastatic deposits, distant from the primary tumour [74].

*E. Coli* is another common organism found to be over-represented on CRC mucosa [75]. *E. coli* promotes tumour growth, both *in vitro* and in a xenograft model, via its genotoxin colibactin [76] and expresses genes that are known to have oncogenic relevance, purporting to M-cell translocatory, angiogenic and genotoxic properties [77]. Both avirulent and pathogenic strains of colonic *E. coli* are able to exert this genotoxic influence via outer membrane vesicles (OMVs) [78]. This is important as the colonisation of *E. coli* grown from colonic mucosa is a poor prognostic factor for colon cancer and it correlates with the TNM stage. Specifically, pathogenic cyclomodulin-positive *E. coli* strains are more prevalent on the mucosa of patients with stages III/IV than those with stage I colon cancer [79].

## Future perspectives: accounting for the complexity of the CRC microbiome

As we have seen, the majority of existing research on the gut microbiota in CRC has fallen into two broad categories: (1) cataloguing exercises which seek to describe colonic ecological composition and thus make inferences about bacteria which may be over-represented in the disease state and (2) reductive science which identifies and thoroughly investigates (using *in vitro* and/or animal models) a particular bacterium (e.g. *F. nucleatum*) with putative pro-neoplastic capabilities. Both of these approaches have their drawbacks. The former, in the absence of complementary techniques such as metabolomics, fails to provide insights on microbiota function. The latter is likely to underestimate the complexity of the diverse microbial communities co-existing in the developing tumour micro-environment [80]. It is perhaps telling that *F. nucleatum*, the pathobiont which has aroused most attention in CRC, appears to be found in only 13% of human CRCs [73]. Moreover, attempts to replicate the findings of earlier studies have demonstrated contradictory results regarding the prevalence and abundance of *F. nucleatum* in CRC [81].

In addition, concerns remain about the validity of existing findings in this field of research. Early studies suffered from a lack of consistency in sampling protocols, differing analytical methodologies and small numbers of patients. There was often a paucity of adequate clinical phenotyping data and an unrealistic supposition that polyps and CRCs are pathologically homogeneous. Although some of these issues are better addressed by contemporary studies [82, 83], the worry lingers that conclusions may be skewed by unappreciated confounding factors, including ethnicity, comorbidity and medication. Furthermore, it is probable that the circumstances in which samples are taken will also influence the ecological characteristics of the CRC microbiome and there remains considerable disagreement about the best methods for sampling the microbiota, with mucosal biopsies, stool and rectal swabs all providing differing information [84, 85]. Finally, the elephant in the room for all cancer microbiome studies is the issue of separating causation from association [86]. The solution to this problem has to include large, prospective, cohort studies with longitudinal sampling of participants prior to the development of the disease, a process that will take decades. The arguments in favour of this approach are being made ever more vociferously [87].

**Figure 2.**
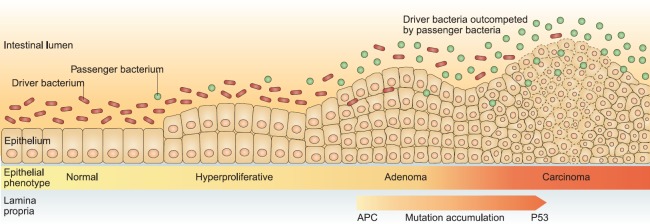
The driver-passenger model. Taken from ‘A bacterial driver-passenger model for CRC: beyond the usual suspects’. Tjalsma et al [88] Nature Reviews Microbiology (2012).

Even with better human studies, given the enormous inter-individual variability and diversity of composition and function of the colonic microbiota, a more sophisticated analytical approach is clearly required. An attempt to conceptualise this complexity is provided by the driver-passenger model [88]. It postulates that genetically susceptible individuals might be colonised by a cocktail of pathogenic bacteria, which can cause inflammation, increase cell proliferation and produce genotoxic substrates, thus driving the initiation of early neoplastic lesions and contributing to the accumulation of genetic mutations as lesions progress (Figure 2). Furthermore, as the tumour develops, with associated changes in the metabolic milieu of the tumour microenvironment, these pathogenic driver species may be out-competed by opportunistic passengers, which thrive in the altered ecological niche. This model too is in all probability overly simplistic: perhaps, there are active passengers, which facilitate local invasion and distant metabolic spread.

Critically, it remains the case that the function and metabolic niche requirements of bacterial communities in diseases such as CRC are largely unknown. A range of ‘omics’ techniques are now being developed to address this knowledge shortfall [89]. These include metatranscriptomics and metaproteomics, which go some way to bridge the gap, but it is metabolomics, which seeks to identify and quantify all of the metabolites present in a sample, that emerges as the most suitable application to integrate with studies of the microbiome [90].

## Conclusion

With advances in computational and systems medicine, investigators are producing novel insights into colonic gene-environment interactions [91] and data are now emerging suggesting that the colonic microbiota plays a vital symbiotic role in determining the metabolic milieu of the tumour micro-environment [92]. We now have the opportunity to mine extremely large parallel sets of clinical, epidemiological, dietary, pathological and omics data to streamline the direction of ongoing cancer and microbiome research. This approach has already borne fruit in the discipline of molecular pathological epidemiology [93, 94]. Future studies must integrate multi-omics human datasets and leverage their discoveries to guide relevant and suitably refined mechanistic investigation.

## Conflicts of interest

The authors declare that they have no conflicts of interest.

## Figures and Tables

**Table 1.** Bacteria with evidence of pro-oncogenic mechanisms in experimental studies of CRC.

Bacterium	Proposed pro-oncogenic mechanisms
*Streptococcus gallolyticus* [95–97]	COX-2 mediated inflammatory response Beta-catenin dependent cell proliferation
*Enterococcus faecalis* [98, 99]	Bystander effect: Induction of mucosal macrophages to produce clastogens that cause DNA damage through free radical and superoxide production
*E. Coli* [76, 78, 100, 101]	Toxin production: genotoxin Colibactin breaks double-stranded DNA Genotoxicity induced by OMVs Depletion of host mismatch repair proteins via bacterially secreted EspF effector protein
*Bacteroides fragilis* [102–105]	Inflammatory stimulus leads to increased reactive oxygen species production and DNA damage resulting from spermine oxidase polyamine catabolism Stat3 activation of mucosal IL-17 response
*Fusobacterium nucleatum* [61, 68, 69, 72]	Modulation of E-cadherin/B-catenin signalling via FadA adhesion Recruitment of proinflammatory myeloid cells conducive to tumour progression Tumour-immune evasion via Fap2 protein inhibition of NK cell cytotoxicity
*Peptostreptococcus anaerobius* [106]	Increased levels of reactive oxygen species promote cholesterol synthesis and cell proliferation
*Helicobacter hepaticus* [107]	Up-regulation of tissue inducible Nitric Oxide Synthase and TNF-alpha
